# Ligand-Induced Dynamics of Neurotrophin Receptors Investigated by Single-Molecule Imaging Approaches

**DOI:** 10.3390/ijms16011949

**Published:** 2015-01-16

**Authors:** Laura Marchetti, Stefano Luin, Fulvio Bonsignore, Teresa de Nadai, Fabio Beltram, Antonino Cattaneo

**Affiliations:** 1 National Enterprise for nanoScience and nanoTechnology (NEST) Laboratory, Scuola Normale Superiore and Istituto Nanoscienze-CNR, Piazza San Silvestro 12, Pisa I-56127, Italy; E-Mails: l.marchetti@sns.it (L.M.); s.luin@sns.it (S.L.); fulvio.bonsignore@sns.it (F.B.); f.beltram@sns.it (F.B.); 2 Biology Laboratory (BioSNS), Scuola Normale Superiore and Istituto di Neuroscienze-CNR, via Moruzzi 1, Pisa I-56100, Italy; E-Mail: teresa.denadai@sns.it

**Keywords:** neurotrophin receptors, nerve growth factor, single molecule imaging, single molecule tracking, single molecule interactions, binding stoichiometry, receptor clustering, membrane trafficking, receptor internalization

## Abstract

Neurotrophins are secreted proteins that regulate neuronal development and survival, as well as maintenance and plasticity of the adult nervous system. The biological activity of neurotrophins stems from their binding to two membrane receptor types, the tropomyosin receptor kinase and the p75 neurotrophin receptors (NRs). The intracellular signalling cascades thereby activated have been extensively investigated. Nevertheless, a comprehensive description of the ligand-induced nanoscale details of NRs dynamics and interactions spanning from the initial lateral movements triggered at the plasma membrane to the internalization and transport processes is still missing. Recent advances in high spatio-temporal resolution imaging techniques have yielded new insight on the dynamics of NRs upon ligand binding. Here we discuss requirements, potential and practical implementation of these novel approaches for the study of neurotrophin trafficking and signalling, in the framework of current knowledge available also for other ligand-receptor systems. We shall especially highlight the correlation between the receptor dynamics activated by different neurotrophins and the respective signalling outcome, as recently revealed by single-molecule tracking of NRs in living neuronal cells.

## 1. Introduction

Neurotrophins (NTs) are a family of neuronal growth factors that crucially regulate cell survival, differentiation, neurite outgrowth, as well as cell maintenance, neurite regeneration and synaptic plasticity in both the central and peripheral nervous system (CNS and PNS, respectively) [1,2]. NTs are synthesized as long precursors (pre-proNTs) that contain a ~2 kDa signal peptide for protein secretion (pre-peptide). The latter is cleaved upon translocation into the endoplasmic reticulum and yields the ~30 kDa precursor proteins (proNTs) that rapidly associate as non-covalent homodimers. The pro-sequence is cleaved at a highly conserved dibasic amino acid site either in the *trans*-Golgi network by furin protease and pro-convertases or, once secreted, in the extracellular space by plasmin or other matrix metalloproteases giving the mature, homodimeric ~26 kDa (13 kDa per monomer) NT [3,4,5,6]. Under physiological conditions, NTs are typically produced and secreted by non-neuronal tissues or by neurons (defined as “targets”) in the PNS and CNS, respectively. When released by postsynaptic targets, NTs bind receptors on nerve terminals of presynaptic NT-sensitive neurons [7,8]. Although recent studies indicate that autocrine and non-target-derived paracrine modes of NT presentation are also likely to be important [7,9,10], target-derived NT actions were the most intensively studied in the latest years. It is now well established that NTs bind specific receptors at the neuronal distal compartments and the activated complexes thereby formed can signal both locally (where they control growth-cone motility) and retrogradely through long axons up to distant cell bodies (where they promote gene expression and survival). Indeed retrograde signalling is the main mechanism regulating the survival/degeneration switch during development and, once started, it must continue for the lifespan of a neuron to maintain its functional differentiated state [7,8,11,12,13,14,15].

**Figure 1:**
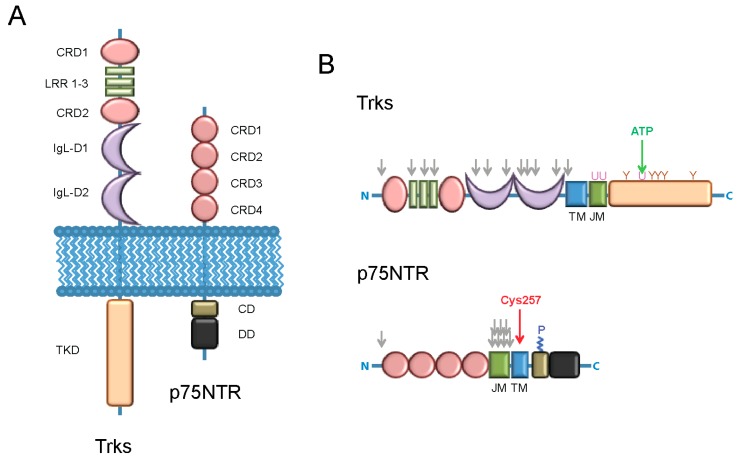
Schematic picture of tropomyosin receptor kinases (Trks) and the p75 neurotrophin receptors (p75NTR). (**A**) Structure of the two receptors: The intracellular (on **top**) and extracellular (on **bottom**) domains are highlighted. The following abbreviations are used: CRD (cystein-rich domain); LRR (leucine-rich domain); IgL-D (immunoglobulin-like domain); TKD (tyrosine-kinase domain); CD (chopper domain); DD (death domain); (**B**) Modified residues of the Trks (on **top**) and of p75NTR (on **bottom**) receptors. The following abbreviations are used: TM (transmembrane domain); JM (juxta-membrane domain); *N* (*N*-terminus); *C* (*C*-terminus). The following symbols are used: Grey ↓ (N- or O-glycosylation sites); green ↓ (ATP-binding site); red ↓ (site of covalent homo-dimerization due to the disulfide bond formed by Cys257 [32,33]). U (ubiquitination-related lysine residues, as derived from studies mainly performed on the TrkA receptor [34,35,36,37]); Y (phosphorylated tyrosine residues, their numeration and function is described in Figure 2); P (palmitoylated Cys residue [38]).

More than fifty years after the discovery of the nerve growth factor (NGF) [16,17], the first and best characterized NT, and thirty years after the discovery of its retrograde transport [18,19], it is still surprising that these factors exert their multitude of biological functions mostly by binding only two types of membrane receptors, hereafter referred to as neurotrophin receptors (NRs): the tropomyosin receptor kinases (Trks) and the p75 neurotrophin receptor (p75NTR) [20,21,22,23,24,25,26]. Figure 1 schematically shows these two NRs and highlights the lack of relation between their structures. Trks belong to the receptor tyrosine kinase (RTK) family: these are traditionally known to dimerize upon ligand binding, thus activating the *trans*-phosphorylation of the intracellular kinase domains, and additional phosphorylation of intracellular effectors; alternatively, there is increasing evidence that many RTK members exist as dimers or clusters also in the absence of ligands, and the dimers can be active, eventually stabilized by ligand binding and/or primed for ligand-induced activation [27]. For the Trks such activation mechanisms ensue in mostly neurotrophic and survival responses [22]. On the other hand, p75NTR is a member of the tumor necrosis factor receptor (TNFR) superfamily: It has no intracellular enzymatic activity and is best known for mediating neural-cell death during development as well as in the adult following injury [25,28,29]. The specification of differential neuron-specific actions is achieved by NT binding to NRs using a number of different but interlaced molecular mechanisms, the main of which will be listed in the following.

Different NTs selectively recognize different Trks; mammals express four NTs, namely NGF, brain-derived growth factor (BDNF), neurotrophin 3 (NT-3) and neurotrophin 4/5 (NT-4/5). They bind specifically to TrkA (NGF), TrkB (BDNF and NT-4) and TrkC (NT-3) receptors. NT-3 also binds to TrkA and TrkB, although with less affinity and activating distinct signalling outcomes [22,23]. Thus, a first level of specificity relies on the different Trk-expression profile in distinct subsets of peripheral and central neurons. Individual neurons may be responsive to more than one NT at a given time or at subsequent times during development [30,31]; importantly, various splicing isoforms of the three Trks were reported that display both altered ligand-binding affinity for the different NTs and subsequent activation of distinct signalling pathways [22,23].

The presence of p75NTR as an alternative and/or cognate NGF receptor greatly influences ligand-binding specificity and the subsequent signalling outcome. The functional interplay between Trks and p75NTR has been debated for a long time. The first pioneering study of the field described that when p75NTR is co-expressed with TrkA, mature NGF binds to the complex with higher affinity than with either receptor alone [39]. Moreover, p75NTR co-expressed with Trks was found to enhance the selectivity of Trk recognition by NTs [40]. Conversely, p75NTR expressed alone was found to induce death upon NGF binding in different cells of the CNS [41,42]. Therefore, as physiologically TrkA and p75NTR co-exist at various reciprocal expression levels in different neurons and during different stages of neuronal development [43,44], the ratio of p75NTR to Trks was linked to the decision between survival and death among NGF-responsive neurons [45]. Accordingly, such ratio was also shown to regulate the number of high-affinity NGF binding sites, which are responsible for a survival-signalling outcome [46]. However, while enhancement of NGF-affinity to TrkA by p75NTR is well documented, the mechanism by which this occurs is not obvious from the analysis of the available crystal-structure data [47,48,49,50,51]. Over the last twenty years, several mechanistic models for the functional complex formed by TrkA with p75NTR upon NGF binding were proposed, the latest of which is the ligand-passing model in which p75NTR first binds to NGF and then releases the ligand to TrkA [51]. This model does not take into account a previous observation that the extracellular ligand-binding domain of p75NTR and therefore NGF binding by p75NTR are not required to create high-affinity NGF binding sites [46]. This evidence was recently corroborated by the observation that a p75NTR intracellular domain fragment formed upon processing by secretase proteases, but not the full-length p75NTR, enhances NGF binding to TrkA [52]. These observations are suggestive of receptor-receptor interactions likely involving the transmembrane and/or cytoplasmic domains of TrkA and p75NTR. In any case, unambiguous evidence for the existence, stoichiometry and nature of a ternary TrkA-NGF-p75NTR complex is still lacking. An additional layer of complexity was added by the discovery that the precursor forms of NTs, proNTs, display much higher affinity for p75NTR than the respective NTs. They induce p75NTR-dependent apoptosis in cultured neurons at a much lower dose compared to the respective NTs and with minimal activation of TrkA-mediated differentiation or survival [53]. It was later discovered that proNGF creates a signalling complex by simultaneously binding to p75NTR and sortilin, a member of the family of Vps10p-domain receptors, with the pro-region making contacts with sortilin and the mature part with p75NTR [53,54,55]. To summarize, it is today well established that although all NTs bind to the p75NTR receptor with the same affinity, the resulting biological outcome depends on the relative Trk/p75NTR expression levels and on the presence of other ligands (especially precursor NTs) and other co-receptors for p75NTR besides Trks, e.g., sortilin [5,20,23,24]. Indeed, proNTs preferentially activate p75NTR to mediate apoptosis while mature forms activate Trks to promote survival.

NT-NR activated complexes formed in various neuronal subtypes assemble into distinct types of endosomes to differentiate signal-transduction pathways. This conclusion was extensively reviewed by Bronfman *et al.* [10] and arises from several independent observations that mutations and/or alterations of the endosome trafficking and sorting properties displayed by specific neuronal subpopulations result in distinct neurodegenerative diseases. According to this hypothesis, also supported by Matusica *et al.* [11], the signalling activity of activated NRs relies not only on the NT-NR molecular complexes formed upon ligand-binding, but also on the fate of such complexes once internalized; in this context, endocytosis and endosome sorting are expected to play a major role.

Many of the signalling cascades, pathways and effectors underpinning the above-described molecular mechanisms were identified: They are beyond the scope of this review and were thoroughly reviewed elsewhere [5,7,9,10,20,21,22,23,24,26,51]. Nevertheless, how different types of NTs and different types of NRs are co-ordinately working to produce variable behaviours in different neuronal cells is still largely unknown. In particular, the nanoscale details of molecular specificity achieved by NRs upon recognition of different available NTs and/or their respective unprocessed forms are poorly understood. This information would likely provide the mechanistic link between the initial lateral movements triggered at the plasma membrane upon ligand binding, their internalization and transport processes, and their respective biological outcome. This in turn would yield a comprehensive understanding of NT function and open the way to the rational design of targeted pharmacological approaches [26]. Here, we focus on recently-developed advanced imaging approaches—with particular emphasis on single-molecule imaging and tracking—applied to the study of the NT-NR dynamic interplay in the physiological context of living cells. We shall discuss the requirements and potential of such approaches, and why they are particularly suited to address questions such as receptor clustering at the cell membrane and binding stoichiometry of molecular complexes. We shall also review to what extent these biophysical tools were so far applied to gain new molecular details about NT trafficking and signaling. In particular, we shall discuss the recent observation made possible by single-molecule tracking of NRs, that a correlation exists between receptor membrane dynamics activated by different NTs and the respective signaling outcome. The dynamic behavior of NRs will also be discussed in comparison to other similar ligand-receptor systems that were also investigated using similar approaches.

## 2. Advanced Live-Cell Imaging Approaches for the Detection of Molecular Interactions and Single-Molecule Dynamics

Progress in fluorescence imaging is enabling the study of biological events with increasingly high detail thanks to novel microscopy techniques that provide imaging and mapping of several dynamic processes at both cellular and molecular level. Concurrently, several fluorescent probes were developed spanning from fluorescent proteins (FPs) and other protein tags, to organic dyes and nanocrystals that enable the noninvasive study of a large number of cellular processes [56,57,58,59,60,61]. Nowadays there is a growing list of fluorescence-imaging approaches that can be considered “advanced” in that they offer resolution close to or beyond the light diffraction limit and monitor dynamics occurring down to the sub-ms time scale [62,63,64,65,66,67]. Among advanced microscopy techniques that have been used to study NRs, we shall distinguish between two approaches that address complementary aspects of their NR dynamics in living cells: (i) Homo- or hetero-interactions involving NRs at the molecular scale; and (ii) membrane or intracellular dynamics and trafficking of NRs in either resting or ligand-stimulated conditions.

Most of the studies concerning molecular interactions occurring amongst NRs used Förster resonance energy transfer (FRET) imaging [68,69,70,71]. This method exploits the non-radiative energy transfer (due to dipole-dipole coupling) from an excited molecular fluorophore (donor) to another one (acceptor) with absorption spectrum at latter partially overlapping the emission of the donor. Donor and acceptor can be the same fluorophore in the case of Homo-FRET. Upon energy transfer, the acceptor becomes excited and it can emit radiation or energy can be dissipated as heat. Since the efficiency of FRET depends on the inverse sixth power of the distance between the donor and acceptor, it yields a detectable signal only when the two fluorophores are at a distance of at most few nanometers. FRET can therefore reveal whether suitably labeled proteins are at a distance that implies their physicochemical interaction. Technological advances in confocal microscopy imaging combined with the availability of genetically-encoded FPs and/or organic or quantum-dot (Qdot) based FRET pairs provide the tools necessary to detect protein interactions in living cells with high spatial and temporal resolution [72,73]. The spectroscopic properties that must be considered when selecting a workable FRET pair include sufficient separation in absorption spectra to avoid cross-excitation of the two fluorophores, overlap between the emission spectrum of the donor and the absorption spectrum of the acceptor to obtain efficient energy transfer, and separation in emission spectra between donor and acceptor to allow independent measurement of the fluorescence signal of each fluorophore. Several imaging techniques were applied to the evaluation of FRET efficiency [69]: Some of them are based on the quantification of donor and/or acceptor fluorescence intensities (e.g., sensitized emission, acceptor photobleaching), others on donor lifetime measurements (the latter decreases upon FRET occurrence). Another possibility is the measurement of FRET from sensitized acceptor depolarization upon donor excitation with polarized light (fluorescence anisotropy imaging). This last approach was used to investigate the dimerization of p75NTR at the cell surface of living cells using EGFP as fluorophore in a Homo-FRET experiment (see Section 3.2 and [33]).

A different possibility to study interactions and clustering events in living cells (and possibly their dynamics) stems from techniques based on the measurement of fluorescence fluctuations whose ancestor is fluorescence correlation spectroscopy (FCS) but which was extended to study fluorescence fluctuations in and between whole images in a time lapse. These techniques typically exploit FP fusions of the protein of interest. One of the most exploited methods is the number and brightness analysis (N&B) [74]. This is based on the possibility of FCS to separate the brightness of a particle from the number of particles present in the observation volume and therefore to determine the degree of aggregation of proteins. This principle was exploited to obtain the N&B of diffusing molecules at each pixel of an image stack obtained with a laser-scanning microscope, so as to map protein-oligomerization states with high spatial resolution both at the cell membrane and inside living cells [75,76,77,78]. Concerning NRs, the N&B method was recently used to investigate the aggregation of intracellular p75NTR expressed in polarized epithelial cells (see Section 3.2 and [79]).

As far as membrane and intracellular dynamics of NRs are concerned, this was studied by two different, complementary imaging approaches: (i) Ensemble or bulk fluorescence imaging and (ii) single-particle (SP) imaging and tracking. Ensemble imaging usually exploits laser-scanning confocal microscopes and typically measurements are performed on a bulk of molecules (most often, proteins) with fluorescent tags in order to describe their average diffusion features. Events like binding and confinement are usually inferred from their impact on diffusion. Fluorescence recovery after photobleaching (FRAP) [80] is the most famous of these techniques. Most typically, the protein of interest is fused to a FP and transiently or stably transfected in cells. A sub-region of the cell in which the fluorescent protein is localized is photobleached and the recovery of fluorescence intensity in the bleached area is followed as a function of time. The kinetics of this recovery is then used to retrieve quantitative information about the speed at which the fluorolabeled species diffuses from other regions of the cell to the photobleached area [80]. A concern that is raised with FRAP is that it is a perturbing method, in that it requires high-intensity illumination to create the photobleached area thus potentially introducing undesirable effects like cell photodamage [81]. Furthermore, it is an averaging method, *i.e.*, the properties of a large number of fluorescent particles are averaged in time and space. In order to overcome these drawbacks, FRAP studies were progressively replaced by single molecule imaging (SMI) approaches. In this case, the position of individual molecules out of a group is followed in time and the individual traced trajectories can be analyzed to extract quantitative information about particle movement. Trajectories can actually be signatures of the mechanism responsible for the observed molecule dynamics: these characteristics made SMI and SP tracking (SPT) appealing techniques for the study of a wide range of dynamic processes in living cells (e.g., membrane lateral movements [82], intracellular transport [83], viral infection [84], cytoskeleton rearrangements [85], nuclear dynamics [86]). We shall focus hereafter on the challenges posed by the study of protein dynamics at the cell plasma membrane.

The application of SMI and SPT to the study of the dynamics of membrane proteins that can rather freely diffuse within the membrane requires a temporal resolution of at least some (tens of) milliseconds in a field of view that should contain a big part of the plasma membrane of the cell. Additionally, sensitivity and signal-to-noise ratio should allow the detection of the signal from individual single fluorophores. This is difficult to obtain through traditional confocal microscopy, but is instead achievable by wide-field microscopy coupled to fast electron-multiplying charge coupled device (EM-CCD) cameras. Often the time-resolved images are acquired from the basal membrane of the cell by means of total internal reflection fluorescence microscopy (TIRFM). This is a wide-field technique in which the collimated light of a laser is incident on the whole field of view at an angle such that it is totally internally reflected at the glass-water interface. As a consequence, only an evanescent wave penetrates the sample volume with an energy density that decreases exponentially moving away from the interface. Fluorophores are excited only in a region of the specimen immediately adjacent to the glass-water interface. This technique drastically decreases the background signal since fluorescence stemming from fluorescent molecules further away from the interface is suppressed. The depth of the illuminated region can be measured by the length parameter of the decreasing exponential function, which depends on the incidence angle of the laser but is usually ~100 nm, encompassing the basal plasma membrane and the cytoplasmic zone immediately adjacent to it.

The choice of the fluorescent labels is very important when planning SMI and SPT measures, since detection of single molecules (SM) requires the use of bright and photostable fluorophores. The GFP fluorophore is widely used in ensemble imaging methods but displays poor photophysical properties at the SM level. Obtaining trajectories from GFP-tagged single proteins is feasible, but they are too short to allow the derivation of robust information about long-term diffusion as well as oligomerization state of the species of interest [87]. Usually labeling for SPT studies is performed with two different approaches. Proteins endogenously present at the plasma membrane can be detected using specific antibodies (or their Fab fragments), which can in turn be detected using secondary antibodies (or their Fab fragments): the primary or (if needed) the secondary antibody is usually conjugated to small organic dyes, gold nanoparticles, or fluorescent quantum dots (Qdots) [88,89,90,91]. If the proteins are membrane receptors, a possible alternative to antibodies is using their specific ligands conjugated to the same kind of probes [92,93,94]. In the second approach, the proteins of interest are fused to so called “chemical tags”. This novel class of protein labeling strategies exploits defined polypeptide sequences that, once fused to the protein of interest, can be post-translationally modified with appropriately derivatized chemical reagents such as biotin or fluorescent dyes [56,58,61,95]. The labeling of most of chemical tags is compatible with living cells; therefore they are increasingly being used for live imaging purposes. Chemical tags are alternatively classified as: (i) Peptide or protein tags, depending on their size; (ii) self-labeling or enzyme-mediated-labeling tags, depending on whether their coupling occurs spontaneously or is enzyme-mediated; (iii) whole-cell-labeling or surface-labeling tags, depending on whether the chemical label is cell-permeable or not; and (iv) covalently or non-covalently conjugable tags, depending on whether the chemical probe can be attached covalently to the tag. As examples, the SNAP-, Halo-, and ACP- tags are protein chemical tags displaying peculiar chemistry of covalent conjugation that have been intensively used in recent years [58]. The use of chemical tags has the great advantage of ensuring a 1:1 stoichiometry between the protein of interest and the label. This is highly desirable when imaging and counting of SMs is needed, but it requires the ectopic expression of the tagged receptor in the cells, which may result in its over-expression and hamper the individual SM detection. Transgene expression should therefore be carefully controlled, e.g., using inducible promoters that can be exploited to avoid this drawback [96].

SPT raw data typically consist of time series of fluorescent spots moving in the imaged area with a frame recording time of the order of ms or µs. These data are analyzed by a two-step procedure that is often semi-automated to handle the great amount (hundreds to thousands) of trajectories. The first step consists in: (i) Identification of spots corresponding to SPs in each frame; (ii) their localization often at sub-diffraction and sub-pixel resolution; and (iii) connection of spots stemming from the same molecule in different frames, in order to build up the trajectory. This is typically performed with *ad hoc* tracking software tools. Today dozens of such software tools are available and were already reviewed [97] and a critical comparison of their output differences and similarities is available [98]. The second step is needed to compute biologically meaningful quantities from the obtained trajectories. Most typically, the first (and sometimes only) step in the quantitative description of a trajectory is the computation of the mean square displacement (MSD) against lag time [82]. If a trajectory is self-similar (*i.e.*, it undergoes only one type of motion), the trend of MSD identifies the type of motion described by the trajectory: Brownian (or purely diffusive), subdiffusive (e.g., localized), superdiffusive (e.g., drifted). Moreover, a linear fit on an appropriate number of the first MSD points yields a good estimate of the apparent diffusion constant *D* on the frame time scale [99]. Other fits can provide good estimates of confinement length [100], velocity of the drifted component of motion [100], the degree of anomalous diffusion [82]. MSD analysis does not always describe SM dynamics accurately. For example, SMs may not undergo a single kind of motion. In this case, two different approaches are used: (i) Estimate of global dynamic parameters from the whole set of trajectories (or from multimodal trajectories) by considering the entire probability distribution of square displacements (PDSD) [101] as a function of lag time; and (ii) classification of the trajectories in different categories that can also allow to monitor how different biologically-relevant treatments affect each diffusive category. Moreover, even within a single trajectory more than one type of dynamics may be present; a method for determining if a trajectory is “self-similar” is the evaluation of the linearity in the “moment scaling spectrum” (MSS) [102] that also provides a more robust estimate of the coefficient of anomalous diffusion; a second method relies on the measurement of possible transient confinement zones (TCZs) *versus* free diffusion zones [103], from which the distribution of times in the two regimes (and therefore the kinetic constants for entering and exiting these zones) can be calculated. The MSS and TCZ analysis algorithms can even be combined to finally split multimodal trajectories into self-similar sub-trajectories or TCZ zones [104]. Another possibility is to monitor by a rolling-window analysis the trend of more than one parameter at a time (e.g., D, MSD curvature and asymmetry of motion [105]) in the same trajectory to detect transitions between different kinds of motion.

From the analysis of the position trajectories it is also possible to quantify a monomer-dimer equilibrium, either by directly determining merging and splitting events between trajectories of different particles [89,92,106] or, when only a part of the “monomers” are labeled, by evaluating shifts in transient *D* within the same trajectory, e.g., by analyzing the cumulative square displacement (CSD) using a two-state hidden Markov model (HMM) [88]. In any case, the most reliable method to determine all oligomeric states displayed by a protein (and the time duration of each state) in living cells is simply the direct analysis of the number of fluorophores in each fluorescent spot [106]. This is feasible when there is a 1:1 fluorophore:monomer stoichiometry and all fluorophores are in identical excitation and emission configurations. In this case one can determine the number of fluorophores in each spot by the intensity of the corresponding signal [106,107]. Some significant examples of the wide range of biological information that can be extracted from SMI-SPT data are reported in Table 1.

## 3. Advanced Imaging Approaches Applied to the Study of NR Dynamics, Trafficking and Signaling

Several microscopy techniques were applied to study the dynamics of fluorescently-derived NRs and/or the respective ligands in living cells. The first reports addressed the intracellular trafficking of recycling or NT-activated NRs in terms of both local endocytosis and long-range axonal transport. Typically, GFP-fusions of NRs and NTs or fluorescent antibodies recognizing the extracellular epitopes of NRs were used for these purposes in time-lapse imaging experiments. The same GFP fusions of NRs were used in several FRET and bulk diffusivity measurements addressing the issue of homo- or hetero- di/oligomeric receptor complexes formation in living cells. More recently the conjugation of NTs or NRs to bright and photostable fluorophores together with the use of high-performance wide-field and video-rate microscopy have opened the way to studies of imaging and tracking of few or SM of the species of interest in living cells. Such approaches make it possible to probe membrane or intracellular diffusion, oligomerization state and axonal transport of NRs with detail down to the nanoscale. How advanced imaging approaches evolved in the study NT-NR intracellular dynamics and the main results obtained will be separately reviewed for the Trks and the p75NTR receptors in the following two sections.

**Table 1:** SMI-SPT analysis approaches to the study of membrane proteins.

Membrane Protein	Host Cell	Labeling Strategy	Applied Trajectory Analysis	Biological Information Extracted	References
EGFR	CHO	Fab conjugated to Qdot	CSD-HMM	The EGFR monomer-dimer equilibrium is characterized: The receptor is found to exist in both states in resting conditions, with dimers primed for ligand binding and signaling.	[88]
CD36	Macrophages	Fab conjugated to Cy3 or Qdot	MSD; Anisotropy of the scatter of particle positions; MSS	A subpopulation of receptors is identified, characterized by diffusion within linear confinement regions governed by cytoskeleton, whose peculiar geometry is found to promote receptor clustering as well as ligand-induced signaling and internalization.	[89]
FPR	CHO	Peptide ligand conjugated to Alexa594	Analysis of spot intensities	The GPCR monomer-dimer equilibrium is characterized with quantitative details: the two-dimensional monomer-dimer equilibrium constant as well as the association and dissociation rate constants are computed.	[92]
AMPA receptors (with GluR2)	Primary hippocampal neurons from rat embryos	0.5-μm latex beads coated with antibodies against GluR2	MSD; TCZ; distance of trajectories from stained synaptic sites and endocytic pits	During maturation or when raising intracellular calcium, the equilibrium between fast diffusion and stationary behavior of AMPA receptors is more and more shifted to the second state. Stationary zones often correlates with synaptic sites but not with clathrin-coated pits targeted by Eps15. It is suggested that diffusion can rapidly regulate receptor numbers at synapses.	[108]
GlyR	Primary spinal cord neurons from Sprague Dawley rat embryos	Biotinylated Fab against GlyR 1 coupled to Streptavidin coated Qdots	MSD fitting for Brownian and confined trajectories; reconstruction of trajectories from Qdot-blinking-induced shorter ones	Cytoskeleton regulates the localization of GlyR and of gephyrin, the core scaffolding protein of inhibitory post-synaptic differentiation. Microtubules control GlyR lateral diffusion in the extra-synaptic membrane, actin at the synapses.	[109]
TrkA	SH-SY5Y	ACP chemical tag conjugated to Qdot or Atto633	Analysis of combined distributions of parameters computed by MSD, MSS, and TCZ, and spot intensities analysis applied to trajectories and subtrajectories	The interplay between TrkA oligomerization states, local diffusivity and degree of anomalous diffusion is investigated as a function of the binding of different ligands, showing that each ligand promotes distinct TrkA trajectory patterns (“ligand fingerprinting effect”, see Section 3.1).	[104]
β1-AR, β2-AR, GABA_B_ receptor	CHO	SNAP chemical tag conjugated to Alexa647	MSD; Analysis of spot intensities	Three different GPCRs are found to exist at very different degrees of oligomerization (monomer-dimer for β1-AR and β2-AR, and dimer-tetramer for GABA_B_). The lifetime of such oligomeric states depend on receptor density but not on agonist stimulation.	[106]
CD59	T24, Ptk2, NRK	Fab/IgG-gold, IgG-Cy3 or IgG-latex beads conjugates	TCZ	CD59 clusters were shown to undergo periods of actin-driven, stimulation-induced transient arrest of lateral diffusion (STALL), *i.e.*, short-lived immobilization events throughout receptor trajectories. STALL events were correlated to the signaling of the receptor, via recruitment of activated PLCγ effector and subsequent IP_3_-Ca^2+^ spike under the STALL area.	[110]
AMPA receptors (with GluR2)	Primary hippocampal neurons from rat embryos	0.5-μm latex beads coated with antibodies against GluR2	MSD; TCZ; distance of trajectories from stained synaptic sites and endocytic pits	During maturation or when raising intracellular calcium, the equilibrium between fast diffusion and stationary behavior of AMPA receptors is more and more shifted to the second state. Stationary zones often correlates with synaptic sites but not with clathrin-coated pits targeted by Eps15. It is suggested that diffusion can rapidly regulate receptor numbers at synapses.	[108]

CD: cluster of differentiation; EGFR: epidermal growth factor receptor; FPR: *N*-formyl peptide receptor; AMPA: α-amino-3-hydroxy-5-methyl-4-isoxazole propionic acid; GluR2: glutamate receptor subunit 2; GlyR: glycine receptor; AR: adrenergic receptor; GABA: γ-aminobutyric acid.

### 3.1. Trk Receptors

The real-time visualization of Trks trafficking and the evaluation of receptor dynamics in response to NT stimulus were addressed by several, complementary approaches. The first reports were based on GFP-fusions of Trks or of their ligands to study the bulk diffusivity of these molecular species in living neuronal cells. A GFP-TrkB construct was used for a FRAP study of axonal transport in DRG neurons grown in compartmented cultures [111]: GFP was photobleached in the soma compartment, and recovery of fluorescence monitored in response to 10 min of BDNF or control stimulation given at the neurite compartment. This study showed that in live neurons TrkB is rapidly and specifically transported to the cell bodies in response to NT stimulation of the neurites. Plasmids expressing BDNF-GFP were also used, in order to study the secretion dynamics of ectopically expressed NTs [112,113]. In another important study, EGFP fusions with TrkA were expressed in PC12 cells investigating both the full-length receptor and several deletion mutants progressively truncated in the intracellular domain (starting from the *C*-terminus). Authors reported the resulting trafficking between cell surface and cell interior in the presence or absence of NGF [114]. It was found that only the full-length protein properly moved among cell surface, plasma membrane ruffles, endocytic vesicles and perinuclear endoplasmic reticulum. Furthermore TrkA intracellular domain was found to contain two different trafficking motifs, one positively and one negatively regulating NGF-induced receptor internalization. Finally the last ~100 amino acids of TrkA *C*-terminus were found to be necessary for the lysosome degradation of NGF-activated receptors. Notably, in the same work confocal time-lapse analysis during NGF-induced differentiation made it possible to detect real-time transport of vesicles carrying activated full-length TrkA in anterograde and retrograde directions and to measure a transport velocity of ~0.5 µm/s in both cases. This recorded velocity is similar to that measured for radioactive iodide-labeled NGF *in vivo* [19,115]. However, as mentioned above, EGFP photo-physical properties make it difficult to resolve individual or few receptors [87,91,116]. The issue of transport was addressed in subsequent works, in which endogenously expressed TrkA was labeled with Qdots conjugated to the RTA anti-TrkA antibody, a known partial agonist for this receptor [117]. This led to the visualization of receptor bidirectional movements in NGF-differentiated PC12 cells [118]. Qdot-RTA-TrkA molecules were found to move in vesicles displaying velocities of the same order of the ones previously reported (~0.24 µm/s), but in this case small clusters of receptors in each tracked fluorescent spot could be distinguished thanks to the brightness and blinking properties of the probe [118]. However, whether these EGFP- [114] or Qdot-TrkA [118] filled vesicles indeed correspond to signaling endosomes could not be conclusively proved in these studies since the neurotrophin itself was not fluorescent. This limitation was overcome with the preparation, validation and use of various labeled-NGF probes to be used in tracking studies in living cells, the main of which are reported in Table 2. Two main strategies were adopted to (fluoro) label NGF: (i) Chemical modification of NGF at free carboxyl groups using reactive biotin- or organic dye-probes; this was the most widely used NT-labeling approach, even if it suffers from the lack of control in the number and type of modified sites of the target proteins; (ii) fusion of the sequence of NGF to that of a chemical tag that will be covalently coupled to the probe of interest. The latter approach has the advantage of ensuring a 1:1 probe-NT stoichiometry.

**Table 2:** List of the main labeled NGF (nerve growth factor) derivatives reported in literature.

NGF Labeling Strategy	Ratio of Probes Per NGF Molecule	Cellular Model Testing NGF Activity	References
^125^I-NGF	n.d.	Sympathetic neurons	[115]
Biotinylated NGF (NGF-b)	~3 biotin molecules per NGF subunit	PC12	[119]
NGF-b/Streptavidin Alexa647	~9 biotins per NGF molecule; 20 nM NGF-b is given to the cells and further detected with Streptavidin Alexa647	PC12 and PC12*nnr5*	[120]
NGF-b/streptavidin Qdot	≤3 biotins per NGF subunit; NGF-b is conjugated to streptavidin-Qdot at a molar ratio of 1 NGF:1 Qdot	PC12	[121,122]
	~1 biotin was bound per NGF molecule; 2nM NGF-b is given to the cells and further detected with 50–500 pM streptavidin-coated Qdot	Differentiated PC12	[123]
	~3 biotins per NGF dimer; NGF-b is conjugated to streptavidin-Qdot at a molar ratio of 1 NGF:1 Qdot	Rat DRG neurons	[83]
Cy3-NGF or Cy3.5-NGF	~1.0–1.1 ratio between fluorophore and NGF	Chick embryonic DRG neurons; PC12	[124,125,126]
Mono-biotinylated NGF via chemical tag	AVI-tag fused at NGF *C*-terminus; NGF-b is obtained transfecting the construct in HEK293FT cells together with the biotinylating BirA enzyme; NGF-b is conjugated to streptavidin-Qdot at a molar ratio of 1 NGF:1 QD	Rat DRG neurons	[127]
	A4-tag fused at NGF *C*-terminus; NGF-b is obtained by labeling the purified protein with CoenzymeA-biotin substrates and PPTase enzyme	PC12	[128]

Qdot: quantum-dot.

The membrane movements of NGF-TrkA complexes and their trafficking upon endocytosis were investigated at the SM level by using fluorescent cyanine derivatives of NGF [124,125]. Cy3-NGF at sub-nM concentration was monitored in the growth cones of chick dorsal root ganglion: within 1 min, only 40 molecules of ligand were found bound to receptors exposed at the growth cone, first moving on the membrane with a diffusion constant of 0.31 μm^2^/s and then shifting to a drifted, actin-driven rearward movement toward the central region of the growth cone at ~4 μm/min rate. Authors suggested that this rearward trafficking movement precedes NGF-receptor complex internalization in the vicinity of the central region of the growth cone. Actin-driven trafficking of the NGF receptor complex was thus proposed to be an essential step for the accumulation and endocytosis of NGF at the growth cone and for the retrograde transport of NGF towards the cell body [124]. In a parallel work, the same probe was used to investigate the spatiotemporal properties of ligand-receptor complexes formed at the plasma membrane of living PC12 cells: Recorded trajectories typically revealed two distinct transient modes of movement, characterized as mobile phase and immobile phase, with abrupt switching between the two. Notably, the immobile fragments of the trajectories were found to correspond to clustered NGF complexes, to depend on the receptor kinase activity and to co-localize with the signaling effectors recruited under the plasma membrane. Therefore NGF immobilization at the cell membrane was related, albeit indirectly, to the start of signal transduction by activated NRs [125]. Alternatively, NGF conjugated to Qdots was widely used for the long-term imaging of ligand-receptor complexes once internalized into cells [83,118,121,123]. These studies were crucial for the understanding of the details of NGF axonal transport in live DRG neurons [83], of NGF bidirectional movements in the neurites of PC12 cells [123], and of NGF-receptor complexes during endocytic trafficking [121]. In the latest work of this series a GFP fusion of TrkA (TrkA-mSEGFP) and a Cy3.5-NGF conjugate were used in imaging experiments in PC12 cells. TrkA-mSEGFP was found to undergo increased directional (both anterograde and retrograde) movements upon NGF application. Interestingly, the average speed of TrkA-mSEGFP associated with Cy3.5-NGF (anterograde velocity peaks: 0.45–1.50 µm/s; retrograde velocity peaks: 0.45–1.23 µm/s) was remarkably higher than that of TrkA-mSEGFP alone (anterograde velocity peaks: 0.30–1.10 µm/s; retrograde velocity peaks: 0.18–0.45 µm/s). Moreover, co-localization of TrkA-mSEGFP with Cy3.5-NGF was found to occur more frequently in retrogradely moving vesicles than in anterogradely moving ones [126]. The intracellular dynamics of NGF-TrkA complexes was therefore rather extensively investigated, but the study of BDNF-receptor complexes is only starting to be addressed. The axonal movements of Qdot-conjugated BDNF after NR-NT internalization was described in living, compartmented hippocampal neurons and showed that BDNF is transported retrogradely with a mean velocity of ~1 µm/s within both endosomes and multi-vesicular body-like structures [129]. More recently, Vermehren-Schmaedick *et al.* [130] used Qdot-conjugated BDNF to study the recycling and intracellular trafficking of internalized BDNF-receptor complexes in the neuronal soma of nodose ganglion sensory neurons. Differently from the reported linear nature of axonal transport, authors found that trafficking of BDNF complexes at the cell soma is extremely heterogeneous. Reported trajectories in this compartment show no apparent end destination, little time-synchrony and high heterogeneity, e.g., sustained rapid motor transport without pause and immobility for minutes. Data are noteworthy in that they suggest that, whatever the origin, the destiny of NT complexes arriving to the cell soma is by no means trivial and that mechanisms leading to gene expression activation by BDNF need be carefully identified.

The combination of bulk, EGFP-based and SM, Qdot/organic dye-based imaging studies yielded valuable information about the intracellular fate of activated NGF receptor complexes. Nevertheless, none of the cited works addressed the molecular details of NT-induced Trks membrane mobility and early steps of receptor internalization at, or close to, the cell membrane. Similarly, the oligomeric state of Trks in response to NT binding was only briefly addressed. The main reasons for this are at least three: (i) By GFP fusion to the TrkA receptor both the intracellular and membrane pools of protein are labeled and discriminating the two of them requires the use of high resolution imaging techniques such as TIRF microscopy [65]; (ii) the GFP fluorophore displays poor photophysical properties at the single-molecule level and tracking of GFP-tagged single receptors is not feasible for long-enough time to retrieve reliable information about the oligomerization state [87]; and (iii) the alternative use of fluorophore-conjugated NGF only allows the study of TrkA receptor in its activated form, likely in the presence of p75NTR co-receptor, so that the net effect of ligand binding on the membrane mobility of a single receptor is simply not possible.

**Figure 2:**
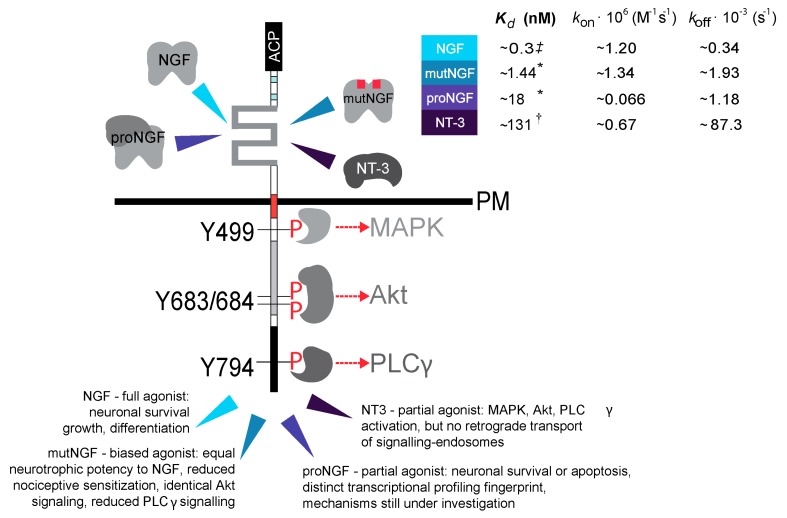
Schematic picture of four different NTs binding to ACP-TrkA. The ACP-TrkA construct and the four ligands investigated for receptor binding [104] are schematically depicted. NGF, NGF R100E mutant (mutNGF, related to HSANV disease [132]), proNGF and NT-3 all bind to the extracellular domain of TrkA receptor but with different affinity, as quantified by the *K_d_* (dissociation constant) values (see the color-coded arrowheads referring to the corresponding *K_d_* values, which are taken from: ***^≠^*** [133]; * [134]; **^†^** [135]). The evoked physiological responses are also different among the four ligands and are summarized at the *C*-terminus of the receptor, highlighted by arrowheads with the same color-code. Intracellular effectors recruited at phosphorylated tyrosine residues and leading to the activation of the MAP kinase, the Akt and PLCγ signaling pathways, upon TrkA-NT binding are also schematically depicted. The numbering of tyrosine residues refers to the rat TrkA cDNA sequence. Note that while Y499 and 794 only have recruitment function, Y683/4 constitute with Y679 (not depicted) the activation loop of tyrosine kinase activity. The figure has been adapted from [104].

In order to avoid these drawbacks, a method was developed that makes it possible to quantitatively investigate the dynamics of individual TrkA receptors in living cells selectively at the cell membrane and independently of its ligand-binding state. In detail, the TrkA cDNA sequence was engineered by adding the ACP (acyl carrier protein) tag sequence [95] to the extracellular domain of TrkA downstream the signal peptide (Figure 2) [100]. More recently, shortened versions of the ACP tag were also inserted that are only 12 amino acids long but yield the same labeling of the full length tag [128]. Together with its cognate PCP (peptidyl carrier protein), the ACP tag is the ancestor of a family of protein and peptide chemical tags, which can be covalently conjugated to virtually any small-probe substituted phosphopantetheinyl (PP) arm of Coenzyme A (CoA) substrate by post-translational modification enzymes named PP transferases (PPTases) [57,61,95]. Some of these may also allow orthogonal labeling of different proteins [128,131]. This tag thus yields site-specific receptor labeling, either with highly stable Qdots or with organic dyes. The ACP-TrkA construct was expressed in TrkA-deficient PC12*nnr*5 cells, it was selectively biotinylated at the cell surface using CoA-biotin substrate by addition of PPTases into the cell medium, and finally conjugated to Streptavidin-coated single Qdots. This strategy made it possible to track the labeled receptors with single-molecule resolution. The tag was shown not to interfere with TrkA receptor function, as the recombinant labeled receptor fully preserves TrkA biochemical and signalling functions, including clathrin-mediated internalization upon NGF addition. Moreover, a preliminary SPT analysis of single fluorescent ACP-TrkAs identified heterogeneous diffusion patterns for this receptor in this cell line [100]. Membrane mobility of labeled ACP-TrkA was also investigated by using TIRF microscopy combined with SPT analysis and the single-molecule dynamics of ACP-TrkA at the membrane of living SH-SY5Y neuroblastoma cells was reported in resting conditions and after binding to four different biologically-relevant ligands for TrkA (see Figure 2 and [104]).

The four investigated ligands (NGF, NGF R100E HSANV mutant, proNGF and NT-3) are all TrkA agonists and display peculiar signaling outcomes [104]. Using Qdot- and Atto633-based receptor labeling for tracking and stoichiometry measurements respectively, it was found that, at low expression levels, most of unstimulated TrkA receptors are fast moving monomers characterized by an average diffusion coefficient of ~0.5 µm^2^/s, about 20% TrkA molecules are moving at least an order of magnitude slower and likely correspond to dimeric and oligomeric states, and around 4% are almost immobile within regions of about 0.6 µm diameter. Ligand binding results in substantial immobilization and slowing down of the receptors, together with an increase of the dimeric and oligomeric populations, consistently with the activated receptor being involved in *trans*-phoshorylation and subsequent interaction with intracellular signaling effectors. Only upon administration of NGF, the fast-diffusive population was observed to consist of monomeric and dimeric forms in dynamic equilibrium, with the latter seemingly moving with almost halved D (~0.3 µm^2^/s) with respect to the former ones; in the same condition, up to 8 TrkA molecules could be counted per slow and confined tracked spots. To be noted that the oligomerization state of the receptors was analyzed using only fluorophores (at a 1:1 stoichiometry with the receptor) at a concentration high enough to achieve almost complete labeling, and all experiments were performed in cells displaying low expression of ACP-TrkA (some units up to few hundreds of molecules on a region in the basal membrane of the order of 10^3^ μm^2^). Crucially, the extent of TrkA lateral mobility modification is strictly ligand-dependent and each ligand promotes distinct trajectory patterns of TrkA receptors at the cell membrane. We called this “ligand-fingerprint effect” and by inducing a set of biochemical modifications of the cell environment, affecting receptor kinase activity, clathrin-coated pits formation, or actin-meshwork integrity, investigated its molecular basis [104]. A close correlation was demonstrated between the initial receptor-membrane dynamics triggered upon ligand binding and the specific biological outcome induced by different ligands for the same receptor.

The observation that NGF addition causes a reduction of the fast diffusing population and a shift of the monomer-dimer equilibrium towards dimeric and clustered forms strongly supports the model of ligand-induced TrkA di-/oligo-merization. Such conclusion may appear in disagreement with data previously reported on the presence of TrkA and TrkB “inactive” dimers independently of NT binding [136,137]. In those articles, the existence of Trks dimers in resting conditions were assessed by using a combination of bimolecular-fluorescence and luciferase-fragment complementation assays of recombinant receptors ectopically expressed, together with chemical crosslinking of cell surface receptors. NT addition was only found to increase dimer phosphorylation, but not the overall dimer population. Since such “inactive” TrkA dimers would not be involved in any interaction with intracellular signaling effectors, one is led to assume that they would be freely moving at the cell membrane. Our analysis shows that if these dimers indeed exist, they are not stable enough to be tracked by our experimental set up, contrary to what happens upon NGF administration. Another possibility is that, at the low receptor expression levels analyzed, we could be abundantly below the dissociation constant (*K_d_*) of the monomer-dimer equilibrium in resting conditions, so that we are able to detect only monomers; upon NGF addition, the *K_d_* becomes lower and a significant amount of fast moving dimers can be detected. Possibly, it could also be that TrkA concentration at the membrane in previous experiments [136,137] was higher than the resting *K_d_*, or the latter was decreased by the used experimental techniques, leading to the detection of preformed dimers. As for slowing-moving dimers and oligomers, they are not only strongly induced by NGF addition but also constitute ≈20% of TrkA trajectories in resting conditions: these may be possibly linked to constitutive internalization and recycling or to transactivation [104]. In any case, it is most likely that some of the discrepancies between the SPT studies and previous cross-linking studies may be explained by the strong dependence of the actual Trks monomer-dimer-oligomer equilibrium either in resting or stimulated conditions, on the receptor expression level as well as by the cell line in which experiments are performed. Indeed, this scenario is well established for the ErbB family (among which, the EGFR was most intensively studied) of RTK: indeed, different advanced-imaging approaches allowed the detection of different receptor-monomer/dimer ratios in resting conditions (see e.g., [88,138,139,140]), depending on the expression level of the investigated receptor. The number of expressed receptors was also linked, together with ligand binding, to the switch from inactive to active EGFR dimers, via a mechanism involving receptor interaction with the cell membrane [141].

### 3.2. p75NTR

Although p75NTR was the first NGF receptor to be identified, and its transport along axons was described before that of Trks [14], studies investigating its intracellular and membrane dynamics using advanced imaging approaches are far less numerous than those reported for the Trks. The earliest articles in the field addressed p75NTR intracellular kinetics and route of NT-induced internalization by confocal microscopy in PC12 cells as well as in motor neurons [120,142]. Live-cell studies were performed using biotinylated NGF (further coupled to fluorescent streptavidin) and/or a fluorophore-conjugated anti-p75NTR antibody recognizing its extracellular domain. These studies established that NGF-p75NTR internalization occurs at a rate approximately three times slower than that of NGF-TrkA complexes in PC12 cells [120]. Interestingly, and differently from TrkA, in motor neurons p75NTR internalization rate was found not to be affected by NT addition to the cell medium; rather, the NT effect was that of re-directing the internalized p75NTR pool from non-coated to clathrin-coated vesicles, which would then be routed to axonal transport [142]. Although sharing the same clathrin-dependent endocytic route, differently from TrkA-filled vesicles, the internalized NTs-p75NTR complexes were observed to be mostly protected from proteolytic degradation and to accumulate in vesicles that did not undergo acidification. These would be later found, both in PC12 cells and sympathetic neurons, to be either recycling endosomes or multivesicular bodies targeted to exosomal release [143]. As these vesicles were found to contain also signaling effectors, the authors finally proposed that NGF-p75NTR signaling endosomes exist, and that such endosomes may be either spatio-temporally and functionally distinct from those containing Trks. The existence of endosomes carrying p75NTR transported by microtubule-associated motors was further proved by the use of a GFP-tagged version of this protein expressed in live cortical and hippocampal neurons. In this case, authors were able to measure a mean velocity of 0.1–0.5 µm/s for anterogradely moving p75NTR vesicles, and of 0.1–1 µm/s for retrogradely moving vesicles [144]. More recently, a Halo tag [145] fusion of p75NTR was reported that was expressed in compartmented cultures of rat sympathetic neurons to monitor its retrograde transport during NGF and BDNF retrograde signaling [146]. The Halo tag belongs to the family of chemical tags [57,61,145] that are preferable to GFP tags for SMI purposes. When fused to recombinant proteins expressed in living cells the Halo tag can be covalently fluorolabeled by adding haloalkane-derivatives of organic dyes into the cell medium. The main advantage of this method is that it allows selective, separate labeling of transfected proteins in the distal axons or cell bodies/proximal axons of compartmented neurons: this makes it possible to independently monitor particles moving along axons in one direction.

Advanced microscopy techniques much helped gaining key insight on p75NTR activation at the cell membrane in response to ligand binding. Vilar *et al.* [33] showed that p75NTR exists both when endogenously or ectopically expressed as pre-formed dimers at the cell membrane independently of ligand binding. The dimer/monomer ratio of cell surface p75NTR was measured by biochemistry methods (biotinylation of cell surface receptors, p75NTR immunoprecipitation, non-denaturing gel electrophoresis and blot) and was shown to vary in different cell types and with different levels of receptor transfected. Such dimeric state was found to rely on the AXXXG motif of the receptor transmembrane domain, and to be crucially stabilized by the formation of a disulfide bond between two cysteine residues belonging to the same domain (Cys257, depicted in Figure 1B). Mutation of Cys257 to alanine did not alter the dimeric state of the receptor but abolished neurotrophin-dependent receptor activity. Authors proposed that these covalent p75NTR dimers are the molecular species able to transduce NT signals. In the same work, FRET experiments were performed with a p75NTR-EGFP construct expressed in living COS-7 cells, in which the degree of receptor self-association was monitored as change of fluorescence anisotropy signal due to homo-FRET between adjacent EGFP fluorophores. These experiments demonstrated a close association of the intracellular domains for both wt (wild-type) and Cys257-mutated p75NTR, consistently with the existence of receptor dimeric forms in the two cases. Intriguingly, NGF addition resulted in a significant increase of anisotropy for the wt but not for the mutant receptor, stemming from a decrease of FRET between two adjacent fluorophores. Authors suggested that rather than a dissociation of p75NTR into monomers (which is not feasible in the scenario of covalently-linked dimers), NTs activate p75NTR by a mechanism involving separation of the intracellular domains of disulfide-linked receptor subunits, with Cys257 acting as the pin of the “dimer scissor” [33]. In a separate work the same authors suggested that such separation of intracellular dimer domains is concomitant to the closure of the dimer extracellular domains upon NT binding [32]. The correlation between p75NTR dimerization and its function, in particular its cleavage by γ-secretase as the final step of regulated intramembrane proteolysis [147,148], was further investigated in living cells by FRET microscopy [149]. In this work, authors set up a sophisticated high-throughput cell-population-based FRET assay with multimode plate readout, using the CFP-YFP FRET pair. The two FPs were fused to a number of different p75NTR constructs, with deletions and/or mutations all along the receptor sequence that were expressed two at a time in HEK293 cells. Measurements were also performed in the presence of p75NTR co-receptors like TrkA, sortilin, and upon addition of NGF or proNGF as ligands. FRET measured for the full-length p75NTR-CFP co-expressed with the respective YFP counterpart was used as an estimate, albeit indirect, of the dimerization degree of the receptor. Authors showed that the transmembrane domain of p75NTR is sufficient for dimerization, which in turn favors the occurrence of regulated intramembrane proteolysis. The structural changes caused or permitted by Cys257-mediated disulfide bond were shown to facilitate γ-secretase cleavage, thus correlating dimer-mediated cell death signaling to the generation of the intracellular domain fragment, as independently assessed by biochemistry assays in neurons [150]. On a side note, authors observed that the co-expression of TrkA, but not sortilin, promotes an increase of p75NTR dimerization, and that TrkA activation promotes the dissociation of p75NTR *C*-terminal fragment dimers through facilitation of γ-secretase cleavage. Finally, and unexpectedly, both NGF and proNGF did not appear to have any influence on the dimerization degree of p75NTR. In conclusion, the NT-induced dynamics of p75NTR appears to be quite different from that of TrkA that is dramatically slowed down upon NGF administration [104,125]. If the interaction of p75NTR with TrkA seems to likely involve their intracellular domains [46,149], this appears not to be the case for p75NTR-sortilin interaction: the same group, using the same cell-population FRET assay, proved that the extracellular stalk JM domain (Figure 1B) is necessary for p75NTR interaction with sortilin [151]. It must be pointed out that all the cited studies provide an indirect estimate of p75NTR dimerization obtained by means of FRET-related techniques. The recent development of p75NTR constructs functionalized with short peptides derived from ACP/PCP tags will allow to directly investigate on the oligomerization state and dynamics of this receptor on the cell membrane, analogously to what previously reported for TrkA [128].

Recently the role of p75NTR dimerization was also studied in a non-neuronal context, *i.e.*, for the case of the apical sorting undergone by this receptor in polarized epithelial cells [79]. In this work, fluorescence fluctuation techniques (photon-counting histogram and N&B analyses [74]) were used to study p75NTR oligomerization status in living cells and to correlate it to receptor polarized localization. Authors found that a GFP fusion of the wt receptor (with EGFP fused at the *C*-terminus of the receptor) forms clusters (up to ~9 receptor molecules per cluster) in the *trans*-Golgi network (TGN) but not at the plasma membrane. Although authors did not investigate further on the p75NTR oligomerization status at the cell surface, they found that the dimerization motif plays a crucial role in inducing receptor clustering, because mutant receptors having dimerization or *O*-glycosylation at the JM domain impaired displayed neither clustering nor selective apical targeting.

## 4. Conclusions and Future Perspectives

The rapid development of advanced fluorescence imaging techniques has required input from biologists and chemists that have provided new fluorescent probes spanning from fluorescent proteins to semiconductor Qdots, and physicists that improved microscope set-ups and analysis software. These contributions led to the unprecedented probing of the nanoscale details of several cellular processes. With these tools in hands, much knowledge was added to the puzzling fate of activated NT-NR complexes within compartments spanning from the neuronal cell membrane to the cell body.

First of all, the details of NR dynamics at the plasma membrane started to be elucidated both in resting conditions and following ligand stimulation. This is an important step towards the complete understanding of the NT-NR interaction network. NT-activated signaling pathways are always the meeting point of several different NTs (and/or their respective precursor forms) binding to different ratios of NRs and/or cognate receptors present at the cell surface of different neuronal cells. Such diversification accounts for the many pathophysiological events in which NTs are involved, but its molecular basis has remained so far unclear. No doubt that a compelling picture of NT function will require inputs from different neuroscience disciplines; nevertheless, we point out here that SMI and SPT approaches applied in turn to the study of the different players involved may allow to identify the molecular species (and related stoichiometry) formed at the cell membrane, which could account for these differential signaling outcomes. Importantly, the behavior of these receptors at the cell membrane represents a first, specific target for pharmacological approaches in a broad range of neurological indications [26]. Thanks to the availability of chemical tags, NRs expressed at the plasma membrane can be labeled with fluorophores that are suitable for SMI purposes so that it is now possible to obtain information about receptor dynamics and oligomeric state at the cell membrane. This led to the observation reviewed here that different NTs binding to TrkA receptor lead to ligand-specific changes of the lateral mobility of single-receptor molecules [104]. Given the sensitivity displayed by the SPT readout of TrkA dynamics upon different stimuli, one can surmise that this assay may be suitable for the screening of compounds selected against this receptor. Furthermore, the “ligand-fingerprint” effect is a significant result to be cast within a broader scenario recently emerged. The specific influence of different ligands for the same receptor was poorly investigated by SMI, as far as receptor dynamics is concerned, and limited to a comparison between agonist and antagonist ligands for the same G protein-coupled receptor [152]. However, specific ligand-receptor signatures, defined as receptor-specific programs of signaling events, were recently reported to regulate different physiological states of phagocytic cells, ultimately leading to the regulation of the whole process of phagocytosis [153]. The discovery of a ligand signature in the receptor membrane dynamics opens the way to several future investigations. Molecular mechanisms linking the different extent of NT-induced receptor clustering and immobilization at the cell membrane to their respective different biological outcomes must be clarified; likely, these involve endosome organization, sorting and trafficking induced by internalized NT-NR activated complexes [11]. Furthermore, it will be interesting to investigate whether the dynamic fingerprint concept can be generalized to other receptors: indeed, there may be a causal correlation between dynamic fingerprints at the cell membrane and the downstream activated signaling pathway. Ligand-induced lateral movements could be conserved or differentiated among different receptors sharing the same signaling pathways, thus providing a molecular key to understand how different growth-factor receptors achieve molecular specificity by using apparently identical signaling cascades.

Although Trks and p75NTR belong to two completely different receptor families, from the work reviewed here they appear to share similar activation events at the cell membrane, among which dimerization is crucial for neurotrophin signaling. In this context, Trks behavior is consistent with that of the other members of the RTK superfamily, such as the ErbB receptors. However, given the diversity displayed by both structure and ligand-binding modes of ErbB and Trk receptors [154], whether the latter display the same inactive-to-active dimer switch as EGFR during ligand-induced activation remains to be established. Different experimental approaches addressing the question of receptor expression level in living cells more systematically must be undertaken. Also, alternative algorithms for SM-trajectory analysis that can detect both short- and long-lived receptor di-/oligo-meric states and possibly link them to their dynamics would much help this analysis; this approach might finally reconcile the two hypotheses of preformed TrkA dimers and of stabilization of dimerization upon ligand binding. These studies should be supported by structural analysis of the re-organization of intracellular domains of the Trks following ligand binding.

p75NTR appears like an anomalous member of the TNFR superfamily that mostly comprises receptors that bind trimeric ligands to induce the trimer- or oligo-merization of their intracellular death domains. p75NTR instead binds to dimeric neurotrophins and its dimerization is functionally significant for neurotrophin signaling. What is still not clear is whether ligand-bound p75NTR dimers undergo clustering at the cell surface, if this depends on available p75NTR co-receptors, and if this is relevant to achieve complex internalization. Furthermore, how the dimeric and possibly oligomeric p75NTR forms relate to the respective NGF-activated TrkA forms at the cell membrane still needs exploration. Indeed, there surely is a differentiation in the intracellular routes undertaken by these two receptors upon activation, proved by their well-established distinct internalization kinetics. Events occurring at the cell membrane and resulting in the dissection of TrkA and p75NTR signaling outcomes constitute an exciting future challenge for SMI of NRs in living cells, and will be likely feasible using a novel imaging toolbox that allows independent and simultaneous tagging of NRs and their ligands [128].

## References

[1] Lewin G.R., Carter B.D. (2014). Neurotrophic Factors.

[2] Skaper S.D. (2012). Neuarotrophic Factors: Methods and Protocols.

[3] Shooter E.M. (2001). Early days of the nerve growth factor proteins. Annu. Rev. Neurosci..

[4] Greene L.A., Shooter E.M. (1980). The nerve growth factor: Biochemistry, synthesis, and mechanism of action. Annu. Rev. Neurosci..

[5] Teng K.K., Felice S., Kim T., Hempstead B.L. (2012). Understanding proneurotrophin actions: Recent advances and challenges. Dev. Neurobiol..

[6] Bruno M.A., Cuello A.C. (2006). Activity-dependent release of precursor nerve growth factor, conversion to mature nerve growth factor, and its degradation by a protease cascade. Proc. Natl. Acad. Sci. USA.

[7] Skaper S.D. (2012). The neurotrophin family of neurotrophic factors: An overview. Methods Mol. Biol..

[8] Ginty D.D., Segal R.A. (2002). Retrograde neurotrophin signaling: Trk-ing along the axon. Curr. Opin. Neurobiol..

[9] Bothwell M. (1995). Functional interactions of neurotrophins and neurotrophin receptors. Annu Rev. Neurosci..

[10] Bronfman F.C., Lazo O.M., Flores C., Escudero C.A. (2014). Spatiotemporal intracellular dynamics of neurotrophin and its receptors: Implications for neurotrophin signaling and neuronal function. Handb. Exp. Pharmacol..

[11] Matusica D., Coulson E.J. (2014). Local *versus* long-range neurotrophin receptor signalling: Endosomes are not just carriers for axonal transport. Semin. Cell Dev. Biol..

[12] Harrington A.W., Ginty D.D. (2013). Long-distance retrograde neurotrophic factor signalling in neurons. Nat. Rev. Neurosci..

[13] Zweifel L.S., Kuruvilla R., Ginty D.D. (2005). Functions and mechanisms of retrograde neurotrophin signalling. Nat. Rev. Neurosci..

[14] Yano H., Chao M.V. (2004). Mechanisms of neurotrophin receptor vesicular transport. J. Neurobiol..

[15] Chowdary P.D., Che D.L., Cui B. (2012). Neurotrophin signaling via long-distance axonal transport. Annu. Rev. Phys. Chem..

[16] Levi-Montalcini R. (1987). The nerve growth factor 35 years later. Science.

[17] Chao M., Cattaneo A., Mobley W. (2013). Rita Levi-Montalcini: The story of an uncommon intellect and spirit. Neuroscience.

[18] Hendry I.A., Stach R., Herrup K. (1974). Characteristics of the retrograde axonal transport system for nerve growth factor in the sympathetic nervous system. Brain Res..

[19] Hendry I.A., Stockel K., Thoenen H., Iversen L.L. (1974). The retrograde axonal transport of nerve growth factor. Brain Res..

[20] Simi A., Ibanez C.F. (2012). Assembly and activation of neurotrophic factor receptor complexes. Dev. Neurobiol..

[21] Schecterson L.C., Bothwell M. (2009). Neurotrophin receptors: Old friends with new partners. Dev. Neurobiol..

[22] Reichardt L.F. (2006). Neurotrophin-regulated signalling pathways. Philos. Trans. R. Soc. Lond. B Biol. Sci..

[23] Chao M.V. (2003). Neurotrophins and their receptors: A convergence point for many signalling pathways. Nat. Rev. Neurosci..

[24] Teng K.K., Hempstead B.L. (2004). Neurotrophins and their receptors: Signaling trios in complex biological systems. Cell. Mol. Life Sci..

[25] Skeldal S., Matusica D., Nykjaer A., Coulson E.J. (2011). Proteolytic processing of the p75 neurotrophin receptor: A prerequisite for signalling?: Neuronal life, growth and death signalling are crucially regulated by intra-membrane proteolysis and trafficking of p75(NTR). Bioessays.

[26] Longo F.M., Massa S.M. (2013). Small-molecule modulation of neurotrophin receptors: A strategy for the treatment of neurological disease. Nat. Rev. Drug Discov..

[27] Casaletto J.B., McClatchey A.I. (2012). Spatial regulation of receptor tyrosine kinases in development and cancer. Nat. Rev. Cancer.

[28] Roux P.P., Barker P.A. (2002). Neurotrophin signaling through the p75 neurotrophin receptor. Prog. Neurobiol..

[29] Underwood C.K., Coulson E.J. (2008). The p75 neurotrophin receptor. Int. J. Biochem. Cell Biol..

[30] Enokido Y., Wyatt S., Davies A.M. (1999). Developmental changes in the response of trigeminal neurons to neurotrophins: Influence of birthdate and the ganglion environment. Development.

[31] Gavazzi I., Kumar R.D., McMahon S.B., Cohen J. (1999). Growth responses of different subpopulations of adult sensory neurons to neurotrophic factors *in vitro*. Eur. J. Neurosci..

[32] Vilar M., Charalampopoulos I., Kenchappa R.S., Reversi A., Klos-Applequist J.M., Karaca E., Simi A., Spuch C., Choi S., Friedman W.J. (2009). Ligand-independent signaling by disulfide-crosslinked dimers of the p75 neurotrophin receptor. J. Cell Sci..

[33] Vilar M., Charalampopoulos I., Kenchappa R.S., Simi A., Karaca E., Reversi A., Choi S., Bothwell M., Mingarro I., Friedman W.J. (2009). Activation of the p75 neurotrophin receptor through conformational rearrangement of disulphide-linked receptor dimers. Neuron.

[34] Arevalo J.C., Waite J., Rajagopal R., Beyna M., Chen Z.Y., Lee F.S., Chao M.V. (2006). Cell survival through Trk neurotrophin receptors is differentially regulated by ubiquitination. Neuron.

[35] Geetha T., Jiang J., Wooten M.W. (2005). Lysine 63 polyubiquitination of the nerve growth factor receptor TrkA directs internalization and signaling. Mol. Cell.

[36] Makkerh J.P., Ceni C., Auld D.S., Vaillancourt F., Dorval G., Barker P.A. (2005). p75 neurotrophin receptor reduces ligand-induced Trk receptor ubiquitination and delays Trk receptor internalization and degradation. EMBO Rep..

[37] Kiris E., Wang T., Yanpallewar S., Dorsey S.G., Becker J., Bavari S., Palko M.E., Coppola V., Tessarollo L. (2014). TrkA *in vivo* function is negatively regulated by ubiquitination. J. Neurosci..

[38] Underwood C.K., Reid K., May L.M., Bartlett P.F., Coulson E.J. (2008). Palmitoylation of the *C*-terminal fragment of p75(NTR) regulates death signaling and is required for subsequent cleavage by γ-secretase. Mol. Cell. Neurosci..

[39] Hempstead B.L., Martin-Zanca D., Kaplan D.R., Parada L.F., Chao M.V. (1991). High-affinity NGF binding requires coexpression of the Trk proto-oncogene and the low-affinity NGF receptor. Nature.

[40] Verdi J.M., Birren S.J., Ibanez C.F., Persson H., Kaplan D.R., Benedetti M., Chao M.V., Anderson D.J. (1994). p75NGFR regulates Trk signal transduction and NGF-induced neuronal differentiation in MAH cells. Neuron.

[41] Frade J.M., Rodriguez-Tebar A., Barde Y.A. (1996). Induction of cell death by endogenous nerve growth factor through its p75 receptor. Nature.

[42] Casaccia-Bonnefil P., Carter B.D., Dobrowsky R.T., Chao M.V. (1996). Death of oligodendrocytes mediated by the interaction of nerve growth factor with its receptor p75. Nature.

[43] Barrett G.L., Bartlett P.F. (1994). The p75 nerve growth factor receptor mediates survival or death depending on the stage of sensory neuron development. Proc. Natl. Acad. Sci. USA.

[44] Wyatt S., Davies A.M. (1993). Regulation of expression of mRNAs encoding the nerve growth factor receptors p75 and TrkA in developing sensory neurons. Development.

[45] Yoon S.O., Casaccia-Bonnefil P., Carter B., Chao M.V. (1998). Competitive signaling between TrkA and p75 nerve growth factor receptors determines cell survival. J. Neurosci..

[46] Esposito D., Patel P., Stephens R.M., Perez P., Chao M.V., Kaplan D.R., Hempstead B.L. (2001). The cytoplasmic and transmembrane domains of the p75 and TrkA receptors regulate high affinity binding to nerve growth factor. J. Biol. Chem..

[47] Gong Y., Cao P., Yu H.J., Jiang T. (2008). Crystal structure of the neurotrophin-3 and p75NTR symmetrical complex. Nature.

[48] He X.L., Garcia K.C. (2004). Structure of nerve growth factor complexed with the shared neurotrophin receptor p75. Science.

[49] Wehrman T., He X., Raab B., Dukipatti A., Blau H., Garcia K.C. (2007). Structural and mechanistic insights into nerve growth factor interactions with the TrkA and p75 receptors. Neuron.

[50] Wiesmann C., Ultsch M.H., Bass S.H., de Vos A.M. (1999). Crystal structure of nerve growth factor in complex with the ligand-binding domain of the TrkA receptor. Nature.

[51] Barker P.A. (2007). High affinity not in the vicinity?. Neuron.

[52] Matusica D., Skeldal S., Sykes A.M., Palstra N., Sharma A., Coulson E.J. (2013). An intracellular domain fragment of the p75 neurotrophin receptor (p75(NTR)) enhances tropomyosin receptor kinase A (TrkA) receptor function. J. Biol. Chem..

[53] Lee R., Kermani P., Teng K.K., Hempstead B.L. (2001). Regulation of cell survival by secreted proneurotrophins. Science.

[54] Nykjaer A., Lee R., Teng K.K., Jansen P., Madsen P., Nielsen M.S., Jacobsen C., Kliemannel M., Schwarz E., Willnow T.E. (2004). Sortilin is essential for proNGF-induced neuronal cell death. Nature.

[55] Feng D., Kim T., Ozkan E., Light M., Torkin R., Teng K.K., Hempstead B.L., Garcia K.C. (2010). Molecular and structural insight into proNGF engagement of p75NTR and sortilin. J. Mol. Biol..

[56] Chen Z., Cornish V.W., Min W. (2013). Chemical tags: Inspiration for advanced imaging techniques. Curr. Opin. Chem. Biol..

[57] Fernandez-Suarez M., Ting A.Y. (2008). Fluorescent probes for super-resolution imaging in living cells. Nat. Rev. Mol. Cell Biol..

[58] Hinner M.J., Johnsson K. (2010). How to obtain labeled proteins and what to do with them. Curr. Opin. Biotechnol..

[59] Pierobon P., Cappello G. (2012). Quantum dots to tail single bio-molecules inside living cells. Adv. Drug Deliv. Rev..

[60] Tsien R.Y. (2005). Building and breeding molecules to spy on cells and tumors. FEBS Lett..

[61] Wombacher R., Cornish V.W. (2011). Chemical tags: Applications in live cell fluorescence imaging. J. Biophotonics.

[62] Cella Zanacchi F., Lavagnino Z., Perrone Donnorso M., del Bue A., Furia L., Faretta M., Diaspro A. (2011). Live-cell 3D super-resolution imaging in thick biological samples. Nat. Methods.

[63] Garcia-Saez A.J., Schwille P. (2007). Single molecule techniques for the study of membrane proteins. Appl. Microbiol. Biotechnol..

[64] Hell S.W. (2003). Toward fluorescence nanoscopy. Nat. Biotechnol..

[65] Kusumi A., Tsunoyama T.A., Hirosawa K.M., Kasai R.S., Fujiwara T.K. (2014). Tracking single molecules at work in living cells. Nat. Chem. Biol..

[66] Levi V., Gratton E. (2007). Exploring dynamics in living cells by tracking single particles. Cell Biochem. Biophys..

[67] Ntziachristos V. (2006). Fluorescence molecular imaging. Annu. Rev. Biomed. Eng..

[68] Elangovan M., Day R.N., Periasamy A. (2002). Nanosecond fluorescence resonance energy transfer-fluorescence lifetime imaging microscopy to localize the protein interactions in a single living cell. J. Microsc..

[69] Ishikawa-Ankerhold H.C., Ankerhold R., Drummen G.P. (2012). Advanced fluorescence microscopy techniques–FRAP, FLIP, FLAP, FRET and FLIM. Molecules.

[70] Sekar R.B., Periasamy A. (2003). Fluorescence resonance energy transfer (FRET) microscopy imaging of live cell protein localizations. J. Cell Biol..

[71] Wallrabe H., Periasamy A. (2005). Imaging protein molecules using FRET and FLIM microscopy. Curr. Opin. Biotechnol..

[72] Albertazzi L., Arosio D., Marchetti L., Ricci F., Beltram F. (2009). Quantitative FRET analysis with the EGFP-mCherry fluorescent protein pair. Photochem. Photobiol..

[73] Marchetti L., Comelli L., D’Innocenzo B., Puzzi L., Luin S., Arosio D., Calvello M., Mendoza-Maldonado R., Peverali F., Trovato F. (2010). Homeotic proteins participate in the function of human-DNA replication origins. Nucleic Acids Res..

[74] Digman M.A., Dalal R., Horwitz A.F., Gratton E. (2008). Mapping the number of molecules and brightness in the laser scanning microscope. Biophys. J..

[75] Di Rienzo C., Jacchetti E., Cardarelli F., Bizzarri R., Beltram F., Cecchini M. (2013). Unveiling LOX-1 receptor interplay with nanotopography: Mechanotransduction and atherosclerosis onset. Sci. Rep..

[76] James N.G., Digman M.A., Gratton E., Barylko B., Ding X., Albanesi J.P., Goldberg M.S., Jameson D.M. (2012). Number and brightness analysis of LRRK2 oligomerization in live cells. Biophys. J..

[77] Ossato G., Digman M.A., Aiken C., Lukacsovich T., Marsh J.L., Gratton E. (2010). A two-step path to inclusion formation of huntingtin peptides revealed by number and brightness analysis. Biophys. J..

[78] Storti B., Bizzarri R., Cardarelli F., Beltram F. (2012). Intact microtubules preserve transient receptor potential vanilloid 1 (TRPV1) functionality through receptor binding. J. Biol. Chem..

[79] Youker R.T., Bruns J.R., Costa S.A., Rbaibi Y., Lanni F., Kashlan O.B., Teng H., Weisz O.A. (2013). Multiple motifs regulate apical sorting of p75 via a mechanism that involves dimerization and higher-order oligomerization. Mol. Biol. Cell.

[80] Reits E.A., Neefjes J.J. (2001). From fixed to FRAP: Measuring protein mobility and activity in living cells. Nat. Cell Biol..

[81] Klonis N., Rug M., Harper I., Wickham M., Cowman A., Tilley L. (2002). Fluorescence photobleaching analysis for the study of cellular dynamics. Eur. Biophys. J..

[82] Saxton M.J., Jacobson K. (1997). Single-particle tracking: Applications to membrane dynamics. Annu. Rev. Biophys. Biomol. Struct..

[83] Cui B., Wu C., Chen L., Ramirez A., Bearer E.L., Li W.P., Mobley W.C., Chu S. (2007). One at a time, live tracking of NGF axonal transport using quantum dots. Proc. Natl. Acad. Sci. USA.

[84] Brandenburg B., Zhuang X. (2007). Virus trafficking-learning from single-virus tracking. Nat. Rev. Microbiol..

[85] Akhmanova A., Steinmetz M.O. (2008). Tracking the ends: A dynamic protein network controls the fate of microtubule tips. Nat. Rev. Mol. Cell Biol..

[86] Morisaki T., Muller W.G., Golob N., Mazza D., McNally J.G. (2014). Single-molecule analysis of transcription factor binding at transcription sites in live cells. Nat. Commun..

[87] Hibino K., Hiroshima M., Takahashi M., Sako Y. (2009). Single-molecule imaging of fluorescent proteins expressed in living cells. Methods Mol. Biol..

[88] Chung I., Akita R., Vandlen R., Toomre D., Schlessinger J., Mellman I. (2010). Spatial control of EGF receptor activation by reversible dimerization on living cells. Nature.

[89] Jaqaman K., Kuwata H., Touret N., Collins R., Trimble W.S., Danuser G., Grinstein S. (2011). Cytoskeletal control of CD36 diffusion promotes its receptor and signaling function. Cell.

[90] Opazo P., Labrecque S., Tigaret C.M., Frouin A., Wiseman P.W., de Koninck P., Choquet D. (2010). CaMKII triggers the diffusional trapping of surface AMPARs through phosphorylation of stargazin. Neuron.

[91] Triller A., Choquet D. (2005). Surface trafficking of receptors between synaptic and extrasynaptic membranes: And yet they do move!. Trends Neurosci..

[92] Kasai R.S., Suzuki K.G., Prossnitz E.R., Koyama-Honda I., Nakada C., Fujiwara T.K., Kusumi A. (2011). Full characterization of GPCR monomer-dimer dynamic equilibrium by single molecule imaging. J. Cell Biol..

[93] Piguet J., Schreiter C., Segura J.M., Vogel H., Hovius R. (2011). Acetylcholine receptor organization in membrane domains in muscle cells: Evidence for rapsyn-independent and rapsyn-dependent mechanisms. J. Biol. Chem..

[94] Winter P.W., van Orden A.K., Roess D.A., Barisas B.G. (2011). Actin-dependent clustering of insulin receptors in membrane microdomains. Biochim. Biophys. Acta.

[95] Johnsson N., George N., Johnsson K. (2005). Protein chemistry on the surface of living cells. Chembiochem.

[96] Suzuki K.G., Kasai R.S., Fujiwara T.K., Kusumi A. (2013). Single-molecule imaging of receptor-receptor interactions. Methods Cell Biol..

[97] Meijering E., Dzyubachyk O., Smal I. (2012). Methods for cell and particle tracking. Methods Enzymol..

[98] Chenouard N., Smal I., de Chaumont F., Maska M., Sbalzarini I.F., Gong Y., Cardinale J., Carthel C., Coraluppi S., Winter M. (2014). Objective comparison of particle tracking methods. Nat. Methods.

[99] Michalet X. (2010). Mean square displacement analysis of single-particle trajectories with localization error: Brownian motion in an isotropic medium. Phys. Rev. E Stat. Nonlin. Soft Matter Phys..

[100] Callegari A., Luin S., Marchetti L., Duci A., Cattaneo A., Beltram F. (2012). Single particle tracking of acyl carrier protein (ACP)-tagged TrkA receptors in PC12*nnr5* cells. J. Neurosci. Methods.

[101] Pinaud F., Michalet X., Iyer G., Margeat E., Moore H.P., Weiss S. (2009). Dynamic partitioning of a glycosyl-phosphatidylinositol-anchored protein in glycosphingolipid-rich microdomains imaged by single-quantum dot tracking. Traffic.

[102] Ewers H., Smith A.E., Sbalzarini I.F., Lilie H., Koumoutsakos P., Helenius A. (2005). Single-particle tracking of murine polyoma virus-like particles on live cells and artificial membranes. Proc. Natl. Acad. Sci. USA.

[103] Simson R., Sheets E.D., Jacobson K. (1995). Detection of temporary lateral confinement of membrane proteins using single-particle tracking analysis. Biophys. J..

[104] Marchetti L., Callegari A., Luin S., Signore G., Viegi A., Beltram F., Cattaneo A. (2013). Ligand signature in the membrane dynamics of single TrkA receptor molecules. J. Cell Sci..

[105] Huet S., Karatekin E., Tran V.S., Fanget I., Cribier S., Henry J.P. (2006). Analysis of transient behavior in complex trajectories: Application to secretory vesicle dynamics. Biophys. J..

[106] Calebiro D., Rieken F., Wagner J., Sungkaworn T., Zabel U., Borzi A., Cocucci E., Zurn A., Lohse M.J. (2013). Single-molecule analysis of fluorescently labeled G-protein-coupled receptors reveals complexes with distinct dynamics and organization. Proc. Natl. Acad. Sci. USA.

[107] Das S.K., Darshi M., Cheley S., Wallace M.I., Bayley H. (2007). Membrane protein stoichiometry determined from the step-wise photobleaching of dye-labelled subunits. Chembiochem.

[108] Borgdorff A.J., Choquet D. (2002). Regulation of AMPA receptor lateral movements. Nature.

[109] Charrier C., Ehrensperger M.V., Dahan M., Levi S., Triller A. (2006). Cytoskeleton regulation of glycine receptor number at synapses and diffusion in the plasma membrane. J. Neurosci..

[110] Suzuki K.G., Fujiwara T.K., Edidin M., Kusumi A. (2007). Dynamic recruitment of phospholipase C gamma at transiently immobilized GPI-anchored receptor clusters induces IP3-Ca^2+^ signaling: Single-molecule tracking study 2. J. Cell Biol..

[111] Watson F.L., Heerssen H.M., Moheban D.B., Lin M.Z., Sauvageot C.M., Bhattacharyya A., Pomeroy S.L., Segal R.A. (1999). Rapid nuclear responses to target-derived neurotrophins require retrograde transport of ligand-receptor complex. J. Neurosci..

[112] Egan M.F., Kojima M., Callicott J.H., Goldberg T.E., Kolachana B.S., Bertolino A., Zaitsev E., Gold B., Goldman D., Dean M. (2003). The BDNF val66met polymorphism affects activity-dependent secretion of BDNF and human memory and hippocampal function. Cell.

[113] Kohara K., Kitamura A., Adachi N., Nishida M., Itami C., Nakamura S., Tsumoto T. (2003). Inhibitory but not excitatory cortical neurons require presynaptic brain-derived neurotrophic factor for dendritic development, as revealed by chimera cell culture. J. Neurosci..

[114] Jullien J., Guili V., Derrington E.A., Darlix J.L., Reichardt L.F., Rudkin B.B. (2003). Trafficking of TrkA-green fluorescent protein chimerae during nerve growth factor-induced differentiation. J. Biol. Chem..

[115] Claude P., Hawrot E., Dunis D.A., Campenot R.B. (1982). Binding, internalization, and retrograde transport of 125I-nerve growth factor in cultured rat sympathetic neurons. J. Neurosci..

[116] Seefeldt B., Kasper R., Seidel T., Tinnefeld P., Dietz K.J., Heilemann M., Sauer M. (2008). Fluorescent proteins for single-molecule fluorescence applications. J. Biophotonics.

[117] Clary D.O., Weskamp G., Austin L.R., Reichardt L.F. (1994). TrkA cross-linking mimics neuronal responses to nerve growth factor. Mol. Biol. Cell.

[118] Sundara Rajan S., Vu T.Q. (2006). Quantum dots monitor TrkA receptor dynamics in the interior of neural PC12 cells. Nano Lett..

[119] Rosenberg M.B., Hawrot E., Breakefield X.O. (1986). Receptor binding activities of biotinylated derivatives of β-nerve growth factor. J. Neurochem..

[120] Bronfman F.C., Tcherpakov M., Jovin T.M., Fainzilber M. (2003). Ligand-induced internalization of the p75 neurotrophin receptor: A slow route to the signaling endosome. J. Neurosci..

[121] Rajan S.S., Liu H.Y., Vu T.Q. (2008). Ligand-bound quantum dot probes for studying the molecular scale dynamics of receptor endocytic trafficking in live cells. ACS Nano.

[122] Vu T.Q., Maddipati R., Blute T.A., Nehilla B.J., Nusblat L., Desai T.A. (2005). Peptide-conjugated quantum dots activate neuronal receptors and initiate downstream signaling of neurite growth. Nano Lett..

[123] Echarte M.M., Bruno L., Arndt-Jovin D.J., Jovin T.M., Pietrasanta L.I. (2007). Quantitative single particle tracking of NGF-receptor complexes: Transport is bidirectional but biased by longer retrograde run lengths. FEBS Lett..

[124] Tani T., Miyamoto Y., Fujimori K.E., Taguchi T., Yanagida T., Sako Y., Harada Y. (2005). Trafficking of a ligand-receptor complex on the growth cones as an essential step for the uptake of nerve growth factor at the distal end of the axon: A single-molecule analysis. J. Neurosci..

[125] Shibata S.C., Hibino K., Mashimo T., Yanagida T., Sako Y. (2006). Formation of signal transduction complexes during immobile phase of NGFR movements. Biochem. Biophys. Res. Commun..

[126] Nomura M., Nagai T., Harada Y., Tani T. (2011). Facilitated intracellular transport of TrkA by an interaction with nerve growth factor. Dev. Neurobiol..

[127] Sung K., Maloney M.T., Yang J., Wu C. (2011). A novel method for producing mono-biotinylated, biologically active neurotrophic factors: An essential reagent for single molecule study of axonal transport. J. Neurosci. Methods.

[128] Marchetti L., de Nadai T., Bonsignore F., Calvello M., Signore G., Viegi A., Beltram F., Luin S., Cattaneo A. (2014). Site-specific labeling of neurotrophins and their receptors via short and versatile peptide tags. PLoS One.

[129] Xie W., Zhang K., Cui B. (2012). Functional characterization and axonal transport of quantum dot labeled BDNF. Integr. Biol. (Camb.).

[130] Vermehren-Schmaedick A., Krueger W., Jacob T., Ramunno-Johnson D., Balkowiec A., Lidke K.A., Vu T.Q. (2014). Heterogeneous intracellular trafficking dynamics of brain-derived neurotrophic factor complexes in the neuronal soma revealed by single quantum dot tracking. PLoS One.

[131] Zhou Z., Cironi P., Lin A.J., Xu Y., Hrvatin S., Golan D.E., Silver P.A., Walsh C.T., Yin J. (2007). Genetically encoded short peptide tags for orthogonal protein labeling by Sfp and AcpS phosphopantetheinyl transferases. ACS Chem. Biol..

[132] Capsoni S., Covaceuszach S., Marinelli S., Ceci M., Bernardo A., Minghetti L., Ugolini G., Pavone F., Cattaneo A. (2011). Taking pain out of NGF: A “painless” NGF mutant, linked to hereditary sensory autonomic neuropathy type V, with full neurotrophic activity. PLoS One.

[133] Paoletti F., Covaceuszach S., Konarev P.V., Gonfloni S., Malerba F., Schwarz E., Svergun D.I., Cattaneo A., Lamba D. (2009). Intrinsic structural disorder of mouse proNGF. Proteins.

[134] Covaceuszach S., Capsoni S., Marinelli S., Pavone F., Ceci M., Ugolini G., Vignone D., Amato G., Paoletti F., Lamba D. (2010). *In vitro* receptor binding properties of a “painless” NGF mutein, linked to hereditary sensory autonomic neuropathy type V. Biochem. Biophys. Res. Commun..

[135] Ivanisevic L., Zheng W., Woo S.B., Neet K.E., Saragovi H.U. (2007). TrkA receptor “hot spots” for binding of NT-3 as a heterologous ligand. J. Biol. Chem..

[136] Shen J., Maruyama I.N. (2011). Nerve growth factor receptor TrkA exists as a preformed, yet inactive, dimer in living cells. FEBS Lett..

[137] Shen J., Maruyama I.N. (2012). Brain-derived neurotrophic factor receptor TrkB exists as a preformed dimer in living cells. J. Mol. Signal..

[138] Nagy P., Claus J., Jovin T.M., Arndt-Jovin D.J. (2010). Distribution of resting and ligand-bound ErbB1 and ErbB2 receptor tyrosine kinases in living cells using number and brightness analysis. Proc. Natl. Acad. Sci. USA.

[139] Endres N.F., Das R., Smith A.W., Arkhipov A., Kovacs E., Huang Y., Pelton J.G., Shan Y., Shaw D.E., Wemmer D.E. (2013). Conformational coupling across the plasma membrane in activation of the EGF receptor. Cell.

[140] Teramura Y., Ichinose J., Takagi H., Nishida K., Yanagida T., Sako Y. (2006). Single-molecule analysis of epidermal growth factor binding on the surface of living cells. EMBO J..

[141] Arkhipov A., Shan Y., Das R., Endres N.F., Eastwood M.P., Wemmer D.E., Kuriyan J., Shaw D.E. (2013). Architecture and membrane interactions of the EGF receptor. Cell.

[142] Deinhardt K., Reversi A., Berninghausen O., Hopkins C.R., Schiavo G. (2007). Neurotrophins Redirect p75NTR from a clathrin-independent to a clathrin-dependent endocytic pathway coupled to axonal transport. Traffic.

[143] Escudero C.A., Lazo O.M., Galleguillos C., Parraguez J.I., Lopez-Verrilli M.A., Cabeza C., Leon L., Saeed U., Retamal C., Gonzalez A. (2014). The p75 neurotrophin receptor evades the endolysosomal route in neuronal cells, favouring multivesicular bodies specialised for exosomal release. J. Cell Sci..

[144] Formaggio E., Cantu C., Chiamulera C., Fumagalli G.F. (2008). p75 neurotrophin receptor distribution and transport in cultured neurons. Neurosci. Res..

[145] Los G.V., Encell L.P., McDougall M.G., Hartzell D.D., Karassina N., Zimprich C., Wood M.G., Learish R., Ohana R.F., Urh M. (2008). HaloTag: A novel protein labeling technology for cell imaging and protein analysis. ACS Chem. Biol..

[146] Mok S.A., Lund K., Lapointe P., Campenot R.B. (2013). A Halo Tag^®^ method for assessing the retrograde axonal transport of the p75 neurotrophin receptor and other proteins in compartmented cultures of rat sympathetic neurons. J. Neurosci. Methods.

[147] Kanning K.C., Hudson M., Amieux P.S., Wiley J.C., Bothwell M., Schecterson L.C. (2003). Proteolytic processing of the p75 neurotrophin receptor and two homologs generates *C*-terminal fragments with signaling capability. J. Neurosci..

[148] Jung K.M., Tan S., Landman N., Petrova K., Murray S., Lewis R., Kim P.K., Kim D.S., Ryu S.H., Chao M.V. (2003). Regulated intramembrane proteolysis of the p75 neurotrophin receptor modulates its association with the TrkA receptor. J. Biol. Chem..

[149] Sykes A.M., Palstra N., Abankwa D., Hill J.M., Skeldal S., Matusica D., Venkatraman P., Hancock J.F., Coulson E.J. (2012). The effects of transmembrane sequence and dimerization on cleavage of the p75 neurotrophin receptor by γ-secretase. J. Biol. Chem..

[150] Kenchappa R.S., Zampieri N., Chao M.V., Barker P.A., Teng H.K., Hempstead B.L., Carter B.D. (2006). Ligand-dependent cleavage of the P75 neurotrophin receptor is necessary for NRIF nuclear translocation and apoptosis in sympathetic neurons. Neuron.

[151] Skeldal S., Sykes A.M., Glerup S., Matusica D., Palstra N., Autio H., Boskovic Z., Madsen P., Castren E., Nykjaer A. (2012). Mapping of the interaction site between sortilin and the p75 neurotrophin receptor reveals a regulatory role for the sortilin intracellular domain in p75 neurotrophin receptor shedding and apoptosis. J. Biol. Chem..

[152] Jacquier V., Prummer M., Segura J.M., Pick H., Vogel H. (2006). Visualizing odorant receptor trafficking in living cells down to the single-molecule level. Proc. Natl. Acad. Sci. USA.

[153] Hoffmann E., Marion S., Mishra B.B., John M., Kratzke R., Ahmad S.F., Holzer D., Anand P.K., Weiss D.G., Griffiths G., Kuznetsov S.A. (2010). Initial receptor-ligand interactions modulate gene expression and phagosomal properties during both early and late stages of phagocytosis. Eur. J. Cell Biol..

[154] Lemmon M.A., Schlessinger J. (2010). Cell signaling by receptor tyrosine kinases. Cell.

